# Perceptions of malaria control and prevention in an era of climate change: a cross-sectional survey among CDC staff in China

**DOI:** 10.1186/s12936-017-1790-3

**Published:** 2017-03-31

**Authors:** Michael Xiaoliang Tong, Alana Hansen, Scott Hanson-Easey, Scott Cameron, Jianjun Xiang, Qiyong Liu, Xiaobo Liu, Yehuan Sun, Philip Weinstein, Gil-Soo Han, Craig Williams, Peng Bi

**Affiliations:** 1 School of Public Health, The University of Adelaide, Level 8, Hughes Building, North Terrace Campus, Adelaide, SA 5005 Australia; 2 State Key Laboratory of Infectious Disease Prevention and Control, Collaborative Innovation Center for Diagnosis and Treatment of Infectious Diseases, National Institute for Communicable Disease Control and Prevention, Chinese Center for Disease Control and Prevention, Beijing, 102206 China; 3 Department of Epidemiology, Anhui Medical University, Hefei, 230032 Anhui China; 4 School of Biological Sciences, The University of Adelaide, Adelaide, SA 5005 Australia; 5 Communications and Media Studies, School of Media, Film and Journalism, Monash University, Clayton, VIC 3800 Australia; 6 School of Pharmacy and Medical Sciences, University of South Australia, Adelaide, SA 5001 Australia

**Keywords:** Climate change, Malaria, Infectious diseases, Imported cases, Capacity building, Perception

## Abstract

**Background:**

Though there was the significant decrease in the incidence of malaria in central and southwest China during the 1980s and 1990s, there has been a re-emergence of malaria since 2000.

**Methods:**

A cross-sectional survey was conducted amongst the staff of eleven Centers for Disease Control and Prevention (CDC) in China to gauge their perceptions regarding the impacts of climate change on malaria transmission and its control and prevention. Descriptive analysis was performed to study CDC staff’s knowledge, attitudes, perceptions and suggestions for malaria control in the face of climate change.

**Results:**

A majority (79.8%) of CDC staff were concerned about climate change and 79.7% believed the weather was becoming warmer. Most participants (90.3%) indicated climate change had a negative effect on population health, 92.6 and 86.8% considered that increasing temperatures and precipitation would influence the transmission of vector-borne diseases including malaria. About half (50.9%) of the surveyed staff indicated malaria had re-emerged in recent years, and some outbreaks were occurring in new geographic areas. The main reasons for such re-emergence were perceived to be: mosquitoes in high-density, numerous imported cases, climate change, poor environmental conditions, internal migrant populations, and lack of health awareness.

**Conclusions:**

This study found most CDC staff endorsed the statement that climate change had a negative impact on infectious disease transmission. Malaria had re-emerged in some areas of China, and most of the staff believed that this can be managed. However, high densities of mosquitoes and the continuous increase in imported cases of malaria in local areas, together with environmental changes are bringing about critical challenges to malaria control in China. This study contributes to an understanding of climate change related perceptions of malaria control and prevention amongst CDC staff. It may help to formulate in-house training guidelines, community health promotion programmes and policies to improve the capacity of malaria control and prevention in the face of climate change in China.

**Electronic supplementary material:**

The online version of this article (doi:10.1186/s12936-017-1790-3) contains supplementary material, which is available to authorized users.

## Background

Climate change is one of the most critical challenges confronting the world today. The process of rapid development and excessive human activities have caused an increase of 0.85 ± 0.2 °C in global mean temperature over the period from 1880 to 2012 [1]. In China, the average surface temperature has increased 1.3 °C over the past 58 years (1951–2008) [4] and rapid economic development has been accompanied by landscape changes, environmental degradation, increased urbanization and frequent natural disasters [2]. Precipitation has declined in southern parts of China over the last century but increased by 22–33% in north-west China [1]. The most recent Intergovernmental Panel on Climate Change report (IPCC Fifth Assessment Report) in 2014 has predicted an increase of 1.1–6.4 °C in the average global land and ocean surface temperature from 1990 to 2100 and about 0.4–6 °C increase is projected for China over the period [1]. Climate change has had a negative influence on the transmission of infectious diseases [1, 3].

**Fig. 1:**
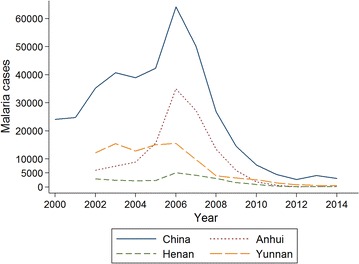
Number of malaria cases in China 2000–2014. Data source was from China National CDC. http://www.chinacdc.cn

One of the concerns of health authorities in China is the possible influence of climate change on vector-borne diseases, especially malaria, a mosquito-borne disease which the authorities aim to eradicate by 2020 [4, 5]. Malaria is a life-threatening parasitic disease, which has been a serious public health problem in China and many other countries. *Plasmodium vivax* and *Plasmodium falciparum* are two predominant species of *Plasmodium* in China [6, 7], and the primary vectors are *Anopheles* mosquito species breeding in the streams, pools, paddy fields and other water bodies [8]. Considerable efforts have been made to address this public health issue in China, which has resulted in a significant decline in its occurrence during the 1980s and 1990s [9]. The reduction in malaria is considered to have been due to exponential improvements in the socioeconomic status and public health interventions [9]. However, early this century there was a re-emergence of the disease over several years [10]. Malaria incidence rapidly increased after 2000 and reached its peak in 2006, during which there were 64,178 cases reported in China [11]. Incidence then decreased markedly between 2007 and 2014 (Fig. 1) when there were 3078 cases reported, most being concentrated in the southwest region—Yunnan Province, central and east region—Anhui, Henan and Jiangsu provinces [6]. Possible reasons for this re-emergence may include meteorological factors, climate change, increased mosquitoes and imported cases, as studies in China have indicated that these factors could have impacts on the transmission of malaria [10, 12–14]. Given the high-density population, rapid economic development, urbanization and increasing numbers of international travelers in China, there is an urgent need to understand the effectiveness of current infectious disease control and prevention of malaria in the face of climate change.

The Center for Disease Control and Prevention (CDC) is an official health agency to protect and improve public health and safety by providing information to enhance health decisions, and promote health through partnerships with provincial health departments and other organizations [15]. One of the significant duties of CDC is infectious disease surveillance, control, and prevention [15]. There are three levels of CDCs in each Province: a provincial-level CDC, prefectural-level CDCs and county/district-level CDCs [16]. The main duties of the provincial-level CDC include the guidance of disease control and prevention across the Province, the provision of professional management, technical support and organizational coordination [15]. Prefectural-level CDCs and county/district-level CDCs are directly responsible for frontline work of infectious disease detection, response, control and elimination, and outbreak investigation in local areas [15]. Consequently, CDC staff are likely to be sensitive to emerging and re-emerging infectious disease threats, and it would be valuable to explore their perceptions of infectious disease control and prevention in the face of climate change.

The aim of this study was to investigate perceptions of CDC staff regarding climate change and malaria control and prevention in China. The findings of this survey highlight the perceptions of China CDC staff of infectious disease control and prevention in the light of climate change. The collective knowledge and experience of the staff may prove useful in preparing for possible increases in malaria and assist policy-makers to formulate adaptation strategies for malaria in China in the context of a changing climate.

## Methods

A questionnaire instrument was administrated to CDC staff in China. Questions were formulated following a review of relevant literature on climate-sensitive diseases in China [17–20]. The full questionnaire explored participants’ beliefs about climate change, which infectious disease(s) would most likely be affected by climate change; perceptions of disease control and prevention; and suggested strategies to strengthen the current capacity to deal with emerging and re-emerging infectious diseases. The questionnaire also included five open-ended response questions, which provided opportunities for participants to comment more openly on infectious disease risk factors. The questionnaire was designed in English and then translated into Chinese. Experts in the China National CDC validated the draft questionnaire, and relevant revisions were made in accordance with recommendations of experts. The questionnaire was then piloted among 18 internal staff in the China National CDC. The results of the pilot testing showed that the questionnaire was clear and well understood. This study reports on a subset of questions relating to malaria and climate change. The relevant section of the questionnaire is included in Additional file 1.

### Study population

**Fig. 2:**
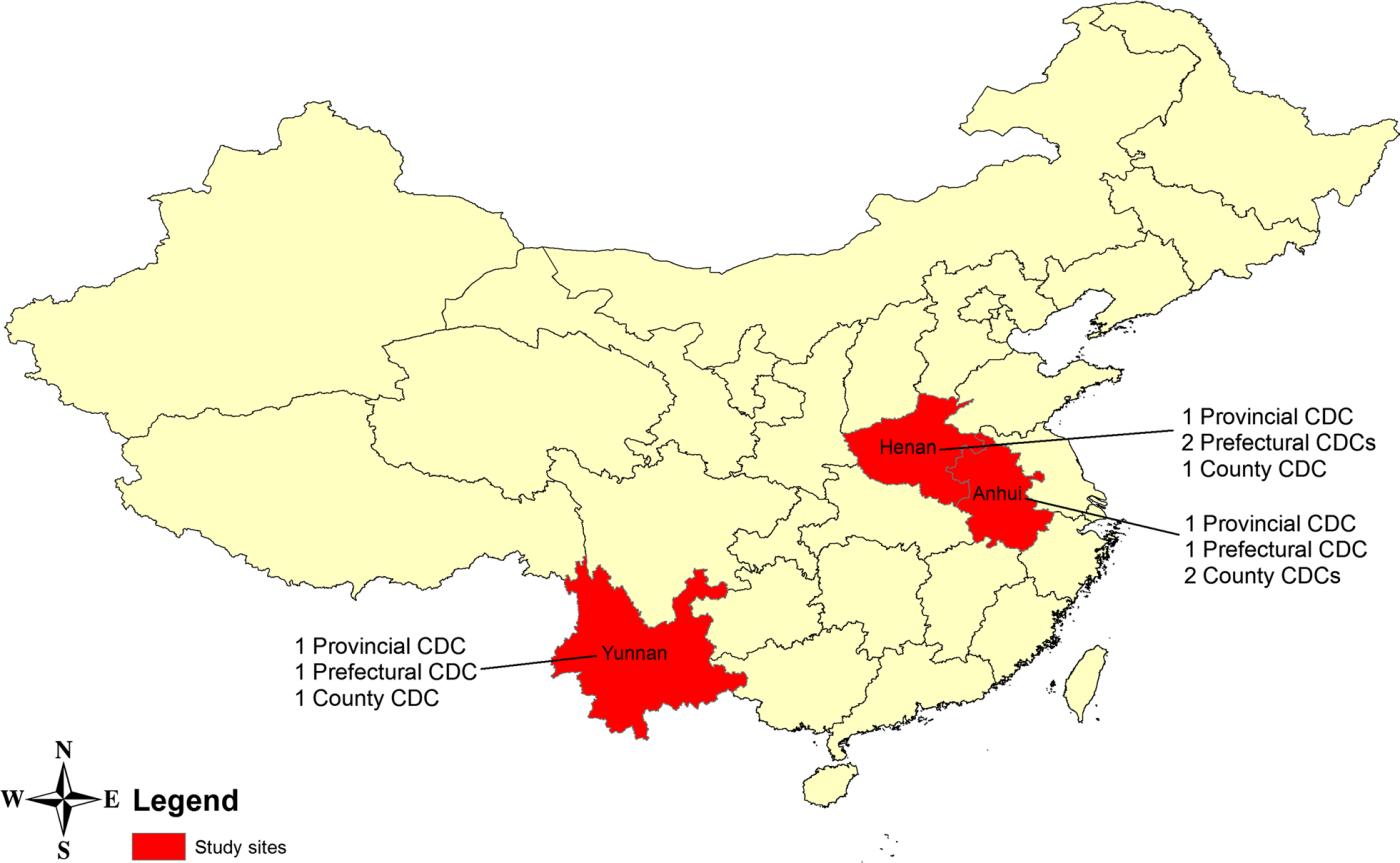
Study sites and sampling CDCs in China

The study population was health professionals in China CDCs whose roles included infectious disease control and prevention, public health, medical laboratory examination and emergency response in their CDCs. Eleven CDCs were involved in this study; three provincial CDCs in Anhui, Henan, and Yunnan Provinces, four prefectural, and county CDCs in the provinces (Fig. 2). The targeted CDCs in Anhui, Henan and Yunnan provinces were selected through discussion with key informants in the China National CDC. These provinces were selected as they had a high incidence of malaria at the time of the study and it was thought that participants could provide informed responses as they had direct experience to deal with malaria.

### Study sites

Covering an area of 9.6 million square kilometres, China has a population of 1.36 billion with several climate zones [21]. Figure 2 shows the study sites.

Anhui Province, located in East China, has a total area of 139,600 km^2^, a population of 60.8 million and GDP of the Province in 2015 was 2200.56 billion Yuan [22–24]. It has a warm-temperate, semi-humid monsoonal climate with an average annual temperature between 14 and 17 °C, and precipitation between 800 and 1800 mm [22]. China’s largest and third largest rivers, Yangtze and Huai, run through the Province [22].

Henan Province, located in Central China, has a total area of 167,000 km^2^, with a population of 106.6 million, is the most populous Province in China [25]. The GDP of the Province in 2014 was 3493.93 billion Yuan [25]. Henan has a temperate climate with four distinct seasons. The average annual temperature is between 10.5 and 16.7 °C, and precipitation varies from 407.7 to 1295.8 mm [25]. China’s second and third largest rivers, Yellow and Huai, run through the Province [25].

Yunnan Province, located in the Southwest of China and bordered by Vietnam, Myanmar, and Laos, has a total area of 394,000 km^2^, a population of 46.8 million and GDP of the Province in 2014 was 1281.45 billion Yuan [26, 27]. It has a sub-tropical highland monsoonal climate. The hottest month is July (19–22 °C) and the coldest is January (6 and 8 °C) [28]. The annual precipitation is above 1000 mm in most areas of Yunnan, and 85% of the precipitation falls between May and October [28]. Six major river systems and many tributaries run through the Province [27].

### Data collection

Three well-trained researchers conducted the fieldwork in China in May 2015. In order to facilitate the questionnaire distribution and to maximize response rates, investigators made contacts with the principal informants working in the selected CDCs and briefly introduced the survey’s aims and main content, then invited them and their colleagues to participate. Participation was voluntary, and no incentives were offered. As responses were anonymous, participants were given clear instructions not to include any personal identifying information on the questionnaire. In total, 624 questionnaires were distributed and later collected from the participating CDCs. All questionnaires were collected, 557 were complete, 67 questionnaires were incomplete. Therefore, the response rate for this study was 89.3%.

### Statistics

Data entry was facilitated using EpiData 3.1 software (EpiData Association, Odense M, Denmark). Stata 13 (Stata Corporation, College Station, Texas, USA) was used to perform all statistical analysis. The demographic characteristics of health professionals were descriptively analysed. The relationship between demographic variables and perception variables was assessed using Chi square test, or Fisher’s exact tests if expected cell frequencies were less than or equal to five [18, 29]. Results were considered statistically significant at a *p* value <0.05.

### Ethics approval

The study was approved by the University of Adelaide (Approval No. HS-2013-052), the University of South Australia (Approval No. 0000032268), Monash University (Approval No. CF13/3263-2013001642), Anhui Medical University (Approval No. 2013007) and the Chinese Center for Disease Control and Prevention (Approval No. ICDC-2013002). Informed consent was obtained from individual participants. This consent procedure was approved by the Chinese Center for Disease Control and Prevention, and conducted in accordance with its guidelines.

## Results

### Demographic characteristics

In this cross-sectional survey, 557 questionnaires were included (268 Males and 275 Females), ranging from 20 to 61 years of age. The demographic characteristics are described in Table 1. One hundred and fifty-four participants were from Anhui CDCs; 231 from Henan CDCs and 172 from Yunnan CDCs. There were 54.8% working in Provincial-level CDC, 27.6% working in Prefectural-level CDC and 17.6% working in District/County-level CDC. With regard to the level of education, the majority of the participants (73.5%) held university degrees. There were 42.6% of the participants who had <10 years working experience at the CDC, and 26.5% had worked for more than 20 years. Junior level staff accounted for 28.7% of the total participants, intermediate level staff 40.9% and senior level staff 19.1%. More than half of the participants (56.8%) undertook duties pertaining to communicable disease control and prevention (including disinfection and vector control, and immunization), 19.3% performed other public health duties (including environmental, occupational, food and school hygiene), 20.0% worked in medical laboratories, and 3.9% worked in emergency response department.

**Table 1:** Demographic characteristics of the participants (N = 557)

Characteristics	Number^a^	Percent (%)
Sites		
Anhui	154	27.6
Henan	231	41.5
Yunnan	172	30.9
Age group (years)		
20–39	301	57.8
≥40	220	42.2
Gender		
Male	268	49.4
Female	275	50.6
Levels of CDC		
Provincial	305	54.8
Prefectural	154	27.6
District/county	98	17.6
Educational level		
Below undergraduate	142	26.5
Bachelor degree	230	42.8
Master degree or above	165	30.7
Length of employment at CDC (years)		
≤9	200	42.6
10–19	145	30.9
≥20	124	26.5
Professional level		
Junior	160	28.7
Intermediate	228	40.9
Senior	106	19.1
Other	63	11.3
Specialty		
Infectious disease control and prevention	235	56.8
Public health	80	19.3
Medical laboratory	83	20.0
Emergency response	16	3.9

^a^The total number may not be equal to 557 for all items as some questions were not answered

### Perceptions of climate change

Table 2 shows the participants’ perception of climate change. The majority (79.8%) were either concerned or very concerned about climate change, and 18.9% were slightly concerned. Furthermore, participants in Henan were the most likely to be concerned about climate change (p = 0.039) (see Additional file 2). In the answer to the question “Do you think your area is becoming warmer?”, 79.7% of participants said “Yes”, 5.0% indicated “No” and 15.3% were “Unsure”. Less than one-third of participants thought they had a good understanding of climate change, and the majority (88%) need more information about the health impacts of climate change. Additionally, male staff appeared to have a better understanding of climate change than female staff (χ^2^ = 5.53, p = 0.019). Staff who had been employed for <10 years were less likely to have a good understanding of climate change (χ^2^ = 9.17, p = 0.01) (see Additional file 2).

**Table 2:** CDC staff’s thoughts on climate change items

Thoughts on climate change items	Number^a^	Percent (%)
How concerned are you about climate change?		
Very concerned	163	29.3
Concerned	281	50.5
Slightly concerned	105	18.9
Not concerned	7	1.3
Do you think your area is becoming warmer?		
Yes	443	79.7
No	28	5.0
Unsure	85	15.3
Do you have a good understanding of climate change?		
Yes	171	31.1
No	379	68.9
Do you need more information about the health impacts of climate change?		
Yes	493	88.7
No	63	11.3

**Fig. 3:**
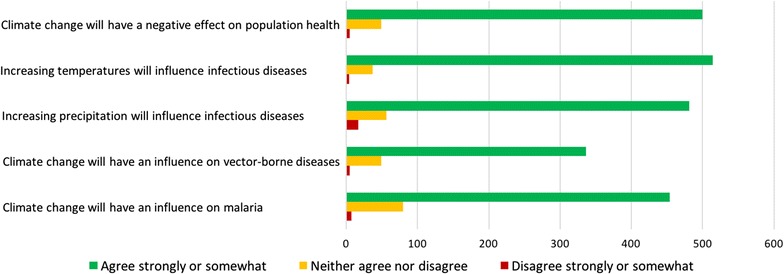
Perception of impacts of public health in the face of climate change

CDC staff’s perceptions of the impact of climate change on public health are summarized in Fig. 3. Overall, 500 participants (90.3%) indicated climate change would have a negative effect on population health, 514 (92.6%) and 481 (86.8%) participants stated that predicted temperature increases and precipitation would influence the transmission of infectious diseases. Most participants (86.1%) agreed that climate change would increase the transmission of vector-borne diseases, and 83.9% expected that climate change would promote malaria transmission. Also, such perceptions were consistent across participants from different CDCs. We also found no significant difference in the perceptions of climate change between these who work in the frontline for disease control and other CDC staff.

### Perceptions of malaria control and prevention

As shown in Table 3, overall, about half (52.4%) of the CDC staff thought the number of mosquitoes had increased over the past 10 years—more in Yunnan, than in Henan and Anhui. There was no significant difference across these three provinces about the participants’ perception (χ^2^ = 4.57, p = 0.335). However, the number of mosquito-borne diseases was perceived to be highest (55.2%) in Yunnan (χ^2^ = 14.49, p = 0.006), and correspondingly fewer participants from Yunnan CDCs claimed there were enough vector control programmes implemented, compared with those from other provinces. About 60% of those from Anhui and Yunnan indicated that mosquito-borne diseases had increased and that this was attributable to climate change while fewer (47.3%) participants from Henan held this opinion.

**Table 3:** Local CDC staff’s perceptions of malaria control and prevention

	Anhui			Henan			Yunnan			Statistics	*p*
	Yes N (%)	Unsure N (%)	No N (%)	Yes N (%)	Unsure N (%)	No N (%)	Yes N (%)	Unsure N (%)	No N (%)		
Is the number of mosquitoes increased?	81 (53.0)	38 (24.8)	34 (22.2)	106 (47.5)	56 (25.1)	61 (27.4)	95 (56.9)	40 (23.9)	32 (19.2)	χ^2^ = 4.57	0.335
Are mosquito-borne diseases increased?	52 (34.2)	53 (34.9)	47 (30.9)	98 (42.8)	69 (30.1)	62 (27.1)	91 (55.2)	41 (24.8)	33 (20.0)	χ^2^ = 14.49	0.006
Would you attribute these to climate change	38 (58.5)	23 (35.4)	4 (6.1)	70 (47.3)	72 (48.6)	6 (4.1)	55 (61.1)	24 (26.7)	11 (12.2)	Fisher’s exact	0.005
Are there vector control programmes in place?	107 (71.3)	31 (20.7)	12 (8.0)	175 (76.4)	47 (20.5)	7 (3.1)	107(64.1)	41 (24.5)	19 (11.4)	χ^2^ = 12.83	0.012
Has malaria re-emerged in this area in recent years?	51 (39.8)	39 (30.5)	38 (29.7)	105 (57.4)	60 (32.8)	18 (9.8)	77 (55.4)	46 (33.1)	16 (11.5)	χ^2^ = 26.24	<0.001
Are some malaria outbreaks occurring in new geographic areas?	21 (17.4)	72 (59.5)	28 (23.1)	65 (35.9)	95 (52.5)	21 (11.6)	51 (36.7)	70 (50.4)	18 (12.9)	χ^2^ = 18.23	0.001
Are some outbreaks occurring at unusual times of the year?	24 (20.0)	71 (59.2)	25 (20.8)	48 (26.8)	99 (55.3)	32 (17.9)	47 (33.8)	76 (54.7)	16 (11.5)	χ^2^ = 8.43	0.077
Have current prevention methods and the National Malaria Programme been effective in reducing incidence in this area?	99 (80.5)	23 (18.7)	1 (0.8)	127 (70.2)	54 (29.8)	0	96 (69.0)	40 (28.8)	3 (2.2)	Fisher’s exact	0.034
Is the population in general well informed about how to reduce the risk of malaria?	86 (70.5)	30 (24.6)	6 (4.9)	118 (64.8)	51 (28.0)	13 (7.2)	81 (58.3)	46 (33.1)	12 (8.6)	χ^2^ = 4.49	0.343

Participants from Anhui Province were less likely to indicate that malaria had re-emerged in their jurisdiction (χ^2^ = 26.24, p < 0.001), than those in Henan and Yunnan province. Regarding the perceived geographical expansion of malaria, about 36% of participants from Henan and Yunnan indicated that they believed malaria had moved into new geographic areas, while significantly fewer (17.4%) participants held this view in Anhui (χ^2^ = 18.23, p = 0.001). Twenty per cent of participants from Anhui indicated that malaria occurrences were arising at unusual times of the year, and more participants in Henan (26.8%) and Yunnan (33.8%) held this opinion. More than two-thirds (73.2%) of participants thought current prevention methods and the National Malaria Programme were effective in reducing the incidence of malaria, and the majority (64.5%) of participants indicated that the local residents in the population were well informed about how to reduce the risk of malaria (Table 3). Moreover, compared with other CDC staff, these who were directly related to the frontline work of disease control CDC staff were more likely to indicate that malaria had re-emerged in their regions in recent years (see Additional file 2).

### Perceptions of the potential main risk factors for malaria

The perceived main risk factors for malaria in China were explored with an open-ended question. Participants were asked ‘What do you think are the main risk factors for malaria in your region?’ Table 4 summarizes these results. The most frequently reported risk factor for malaria in China was mosquitoes in high-density (23.5%), and 21.9% of participants believed imported case of malaria from overseas was a risk factor. Furthermore, 12.8% of participants implicated climate change, 10.6% thought poor environmental conditions, and 8.8% indicated internal migrant population and lack of health awareness as other significant risk factors for malaria. In addition, 12% of participants also thought insufficient control measures, numerous mosquito breeding sites, poor living conditions, poor management of imported cases and geographical factors could contribute to the transmission of malaria.

**Table 4:** Potential main risk factors of malaria

Potential main risk factors	Frequency	Percent (%)
Mosquitoes in high-density	71	25.1
Imported cases	62	21.9
Climate change	36	12.8
Poor environmental conditions	30	10.6
Internal migrant population	25	8.8
Lack of health awareness	25	8.8
Others	34	12.0

Risks were explored by open-ended questions. Keywords of risks were listed in the table. The frequency did not equal to total 557 as some participants either did not answer the questions or answered unclear/unsure about the potential main risk factors

## Discussion

This is the first study to explore CDC staff’s views and understanding of climate change and the transmission of malaria in China. Results showed most participants in our survey were either concerned (50.5%) or very concerned (29.3%) about climate change, and about 19% of participants were slightly concerned. The majority (79.7%) of participants indicated they thought the weather was becoming warmer. However, perceived knowledge of climate change was limited. Less than one-third of participants (31.1%) reported they had a good understanding of climate change and nearly 90%, especially females and new staff, indicated they need more information about climate change impacts. Further in-house training activities could be promoted to improve staff’s knowledge and understanding of climate change. This is consistent with other studies highlighting the need for training and education among CDC staff [29–31].

The vast majority of participants indicated climate change would have an adverse impact on population health, and increase the transmission of infectious diseases, which is line with other studies [32–34]. In particular, 92.6 and 86.8% of participants indicated increasing temperatures and rainfall would influence infectious disease transmission. Furthermore, over 80% of participants believed that climate change would affect vector-borne diseases, including the transmission of malaria. These findings suggest that more attention needs to be paid to malaria control and prevention in the face of climate change to meet the goal of the China National Malaria Elimination Programme by 2020 [5].

About half of the participants indicated they thought the number of mosquitoes had increased over the past 10 years in their regions, despite some vector control programmes being implemented in place. This increase could be due to different reasons, such as urbanization, landscape changes, and the reduction of biodiversity [35]. It may also indicate that current vector control programmes need to be further improved. Some participants stated that mosquito-borne diseases had increased over the period, and this perception was highest in Yunnan Province compared to the other two provinces. This could be due to hotter, more humid weather in Southwest China and numerous imported cases from neighbouring epidemic countries such as Vietnam, Myanmar, and Laos [36, 37], suggesting regional cooperation such as data sharing and information exchange is imperative for malaria and other vector-borne diseases control. Among those participants who believed mosquito-borne diseases had increased, more than half considered the increase was associated with climate change. Studies have found that climate change-induced warmer weather accelerates mosquito life cycles, hastens the development of the malaria parasite within the vector, and contributes to mosquito breeding and biting, and thus disease transmission [10, 12, 38]. Furthermore, warmer weather may have also changed human behavior that could contribute to disease transmission [38].

More than half the participants from Henan and Yunnan CDCs indicated malaria had re-emerged in recent years. This may be due to the increased number of imported malaria cases from overseas (foreign nationality or Chinese workforce infected overseas) and the risk of transmission of infected individuals present to the local population [6, 7]. Furthermore, more participants in Henan and Yunnan thought malaria was occurring in new geographic areas, which are consistent with other studies showing that malaria was emerging and re-emerging in these regions [8, 36, 37]. Greater prevention and control efforts should aim to inhibit malaria transmission in these areas. Although other studies also found malaria had re-emerged in Anhui Province since 2000 [10, 39], participants from Anhui CDCs in this survey were less likely to claim that malaria re-emergence and geographic expansion had occurred. This could be due to the great reduction of malaria cases in Anhui Province from 34984 in 2006 to only 144 in 2014 [11]. Hence, CDC staff in Anhui may be less likely to have observed that malaria had re-emerged compared with the other two Provinces. In addition, the current National Malaria Programme and other related prevention and control measures were thought to be effective in reducing malaria incidence by most of the participants in this survey, which could affirm the overall effectiveness of current control plans. However, in other regions such as Yunnan there may be a need for plans to be further improved to meet the aim of malaria elimination. Furthermore, it will be interesting to know the reasons for the different perceptions of the CDC staff between Henan and Anhui Provinces in terms of malaria emergence and re-emergence, and expansion of epidemic foci, because the two Provinces, especially the malaria epidemic foci are next to each other. In addition, frontline CDC staff were more likely to indicate that malaria re-emerged which could be due to the fieldwork experience they had, compared with other workers. More relevant research among frontline staff in the prefecture and county lower-level CDCs should be conducted to assess the re-emergence of malaria in the context of climate change in China.

The perceived risk factors yielded from open-ended questions are consistent with other studies showing risk factors associated with emergence and re-emergence of malaria in China [35, 38, 40, 41]. High densities of infected *Anopheles* mosquitoes would significantly contribute to the transmission of malaria, and this has been considered one of the most important factors leading to increased incidence of malaria worldwide [38, 40]. Moreover, CDC staff indicated that malaria control and prevention has been severely jeopardized by the growing number of imported cases of malaria, which has also been highlighted by other studies [42–44]. This trend of imported malaria to China has increased gradually since 2006. In 2014, the imported cases of malaria accounted for 98.1% of all malaria cases in China [6]. This implies that action is needed to curb the increasing numbers of imported cases by warning outgoing travelers to avoid potential risk areas or take necessary preventive measures, screening incoming international travelers and returning Chinese workers from overseas to detect and treat cases, and identify types of imported cases [6]. In addition, regional cooperation at the policy level, especially between China and its neighbouring countries, such as Vietnam, Myanmar, and Laos, is very important, in terms of establishment of a similar surveillance system for information exchange, regional collaboration for vector control [45]. Furthermore, more attention to climate change, environmental improvement, internal migrant populations, and health education programmes would also be necessary to reduce the incidence of malaria in China.

This study has several limitations. Firstly, the study participants were from 11 selected CDC, and the result may not be entirely representative of all CDC staff. Secondly, although the study started with a relatively large number of health professionals, the county-level and prefectural-level CDC sample was comparatively small compared with the provincial-level CDC. Future studies could enlarge the samples to recruit more county-level and prefectural-level CDC staff who are working on the frontline for disease control and prevention. Thirdly, the questionnaire survey was conducted in Chinese, and there may be some minor anomalies in translation. Fourthly, response biases may happen as participants may overestimate or underestimate their knowledge and competence. Furthermore, as malaria cases have decreased in China between 2007 and 2014 (Fig. 1) the terms “re-emerged” and “in recent years” in the questionnaire could be viewed subjectively by participants and could be taken to mean the last 15 years or since the 2006 peak. Nevertheless, this study represents health professionals’ perception of infectious disease control and prevention in the face of climate change and also indicates a significant finding that CDC staff may benefit from more scientific information on climate change that, in turn, may help improve the capacity for malaria control and prevention in the face of climate change in China.

## Conclusions

This study found most CDC staff endorsed the statement that climate change has had a negative impact on infectious disease transmission. Malaria is reportedly re-emerging in some areas. However, high densities of mosquitoes and the continuous increase in imported cases of malaria in local areas are critical challenges to malaria control. Further efforts in mosquito density control, imported case surveillance and management, regional cooperation for malaria control and information sharing, climate change-related research and monitoring of potential re-emerging malaria areas are urgently needed. Additionally, comprehensive response measures considering issues of urbanization, internal migrant population, working and living conditions and optimized health promotion strategies are likely to be fruitful in building the capacity of infectious disease control to curb the possible health impact of emerging and re-emerging malaria in China.

## Additional files

**Additional file 1.** Questionnaire.

**Additional file 2.** Supplementary tables.

## Abbreviations

CDC: Center for Disease Control and Prevention
IPCC: Intergovernmental Panel on Climate Change
