# Supplementary material for: Perceptions of malaria control and prevention in an era of climate change: a cross-sectional survey among CDC staff in China
Source: Malar J. 2017 Mar 31;16:136. doi: 10.1186/s12936-017-1790-3 (PMC5374624; doi:10.1186/s12936-017-1790-3)
Supplement: Supplementary file 1 — Additional file 1. Questionnaire. [file 12936_2017_1790_MOESM1_ESM.docx]

**Building capacity to curb the public health impact of emerging and**

**re-emerging infectious diseases due to climate change in China**

**Questionnaire**

Thank you for completing this questionnaire, your help in this research is much appreciated. You may choose to answer all of some of the questions. Your answers will remain anonymous. There are 4 parts to this questionnaire and it should take no longer than 30 minutes to complete:

Part A – Climate change

Part B - Future infectious disease risks in a changing climate

Part C – Capacity building to deal with disease risks

Part D – Demographics

**Part A – Climate change**

**The following questions are about your thoughts on climate change: *(Please indicate your answer with a tick 🗸)***

|  |  | Very concerned |  | Concerned |  | Slightly  Concerned |  | Not concerned |
| --- | --- | --- | --- | --- | --- | --- | --- | --- |
|  | How concerned are you about climate change? |  |  |  |  |  |  |  |
|  |  |  |  |  |  |  |  |  |
|  |  | Yes |  | No |  | Unsure |  |  |
|  | Do you think your area is becoming warmer? |  |  |  |  |  |  |  |

|  | Agree strongly |  | Agree somewhat |  | Neither agree nor disagree |  | Disagree somewhat |  | Disagree strongly |  |  |
| --- | --- | --- | --- | --- | --- | --- | --- | --- | --- | --- | --- |
|  | I think climate change will have a negative effect on population health | |  |  |  |  |  |  |  |  |  |
|  | | |  |  |  |  |  |  |  |  |  |
|  | Predicted increasing temperatures will influence the transmission of infectious diseases | |  |  |  |  |  |  |  |  |  |
|  |  | |  |  |  |  |  |  |  |  |  |
|  | Predicted increasing precipitation will influence the transmission of infectious diseases | |  |  |  |  |  |  |  |  |  |
|  |  | |  |  |  |  |  |  |  |  |  |
|  | I have a good understanding of climate change | |  |  |  |  |  |  |  |  |  |
|  |  | |  |  |  |  |  |  |  |  |  |
|  | I feel I need more information about the health impacts of climate change | |  |  |  |  |  |  |  |  |  |

**Part B - Future Infectious Disease Risks in a Changing Climate**

**B1 Which infectious diseases do you think climate change will affect most in your area? (Tick *🗸* the relevant box for each type)**

|  | Agree strongly |  | Agree somewhat |  | Unsure/ Don’t know |  | Disagree somewhat | |  | | Disagree strongly |
| --- | --- | --- | --- | --- | --- | --- | --- | --- | --- | --- | --- |
| *Vector-borne diseases* |  |  |  |  |  |  | |  | |  |  |
| Malaria |  |  |  |  |  |  | |  | |  |  |

**B2** **In your jurisdiction (province/city/county) over the past ten years has there been:**

|  | | Yes |  | No |  | Unsure |
| --- | --- | --- | --- | --- | --- | --- |
|  | Increases in *mosquito-borne* diseases? |  |  |  |  |  |
|  |  |  |  |  |  |  |
|  | If yes, would you attribute these to climate change? |  |  |  |  |  |
|  |  |  |  |  |  |  |
|  | Increases in mosquito numbers? |  |  |  |  |  |
|  |  |  |  |  |  |  |
|  | Vector control programs in place? |  |  |  |  |  |
|  |  |  |  |  |  |  |

**B4 The next few questions are specifically about malaria *(if there is no malaria in your jurisdiction, please move onto the next question)***

|  | Agree strongly |  | Agree somewhat |  | Neither agree nor disagree |  | Disagree somewhat |  | Disagree strongly |
| --- | --- | --- | --- | --- | --- | --- | --- | --- | --- |
| Malaria has re-emerged in this area in recent years |  |  |  |  |  |  |  |  |  |
|  |  |  |  |  |  |  |  |  |  |
| Some of the malaria outbreaks are occurring in new geographic areas |  |  |  |  |  |  |  |  |  |
|  |  |  |  |  |  |  |  |  |  |
| Some of the outbreaks are occurring at unusual times of the year |  |  |  |  |  |  |  |  |  |
|  |  |  |  |  |  |  |  |  |  |
| The population in general is well informed about how to reduce the risk of malaria |  |  |  |  |  |  |  |  |  |
|  |  |  |  |  |  |  |  |  |  |
| Current prevention methods and the National Malaria Program have been effective in reducing incidence in this area |  |  |  |  |  |  |  |  |  |
|  |  |  |  |  |  |  |  |  |  |
| What do you think are the main risk factors for malaria in your region? *(give details)* |  |  |  |  |  |  |  |  |  |

**Part D – Demographics**

**These final questions are about you and your work:**

Gender: Age: _____________ years

| Male |  |
| --- | --- |
| Female |  |

Affiliation

Institution: ____________________________________________________________

Classification: *(please tick)*

| Junior |  |
| --- | --- |
| Intermediate |  |
| Associate senior |  |
| Senior |  |

Other (*details)* ____________________

Current professional field: *(please tick whichever applies)*

| Nursing |  |
| --- | --- |
| Clinical medicine |  |
| Communicable diseases control and prevention |  |
| Food-borne disease control |  |
| Vector-borne disease control |  |
| Chronic disease control and prevention |  |
| Endemic disease control and prevention |  |
| Emergency response and preparedness |  |
| Laboratory Science/analysis |  |
| Disinfection and vector control |  |
| Immunization planning programs |  |
| Environmental health |  |
| Nutritional health and food safety |  |
| School health |  |
| Occupational health |  |
| Research |  |
| Administrative management |  |
| Informatics and data management |  |
| Health assessment |  |
| Preventive care |  |

Other: *(details)* ____________________________________

How long have you been employed in this profession? _______________ years

Qualifications:

Highest educational qualification:

| Junior high school |  |
| --- | --- |
| Senior high school |  |
| Technical secondary school |  |
| College |  |
| University bachelor’s degree |  |
| Master’s degree or equivalent |  |
| Doctor of Philosophy |  |
