# Supplementary material for: Perceptions of malaria control and prevention in an era of climate change: a cross-sectional survey among CDC staff in China
Source: Malar J. 2017 Mar 31;16:136. doi: 10.1186/s12936-017-1790-3 (PMC5374624; doi:10.1186/s12936-017-1790-3)
Supplement: Supplementary file 2 — Additional file 2. Supplementary tables. [file 12936_2017_1790_MOESM2_ESM.docx]

**Table S1.** Respondents at different CDCs and their concerns about climate change

| **CDC** | | **How concerned are you about climate change?** | | | | | | **Total** | **Statistics** | ***p*** |
| --- | --- | --- | --- | --- | --- | --- | --- | --- | --- | --- |
|  |  | **Very concerned** | **Concerned** | | **Concerned a little** | | **Not concerned** |  |  |  |
|  | Anhui CDCs | 40 | | 81 | | 31 | 2 | 154 |  |  |
|  | Henan CDCs | 84 | | 111 | | 33 | 3 | 231 | Fisher’s exact | 0.039 |
|  | Yunnan CDCs | 39 | | 89 | | 41 | 2 | 171 |  |  |
|  | Total | 163 | | 281 | | 105 | 7 | 556 |  |  |

**Table S2.** Respondents’ perceptions of their understanding of climate change

| **Variables** | | **Do you have a good understanding of climate change?** | | | **Total** | **χ^2^** | ***p*** |
| --- | --- | --- | --- | --- | --- | --- | --- |
|  |  | **Yes** | **No** | |  |  |  |
| Gender | |  |  |  |  |  |  |
|  | Male | 96 | 170 | | 266 |  |  |
|  | Female | 72 | 198 | | 270 | 5.5295 | 0.019 |
|  | Total | 168 | 368 | | 536 |  |  |
| Length of employment (Years) | |  |  |  |  |  |  |
|  | ≤9 | 49 | 149 | | 198 |  |  |
|  | 10-19 | 42 | 101 | | 101 | 9.1717 | 0.010 |
|  | ≥20 | 50 | 73 | | 123 |  |  |
|  | Total | 141 | 323 | | 464 |  |  |

**Table S3.** Comparison of frontline CDC staff’s and non-frontline CDC staff’s perceptions of malaria control and prevention

| **Frontline CDC staff** | | **Malaria has re-emerged in this area in recent years** | | | **Total** | **χ^2^** | ***p*** |
| --- | --- | --- | --- | --- | --- | --- | --- |
|  |  | **Yes** | **Unsure** | **No** |  |  |  |
|  | Yes | 59 | 5 | 20 | 84 |  |  |
|  | No | 174 | 67 | 125 | 366 | 15.5824 | 0.000 |
|  | Total | 233 | 72 | 145 | 450 |  |  |
